# Preparation, Features, and Efficiency of Nanocomposite Fertilisers Based on Glauconite and Ammonium Dihydrogen Phosphate

**DOI:** 10.3390/ma16186080

**Published:** 2023-09-05

**Authors:** Maxim Rudmin, Boris Makarov, Adrián López-Quirós, Prokopiy Maximov, Valeria Lokteva, Kanipa Ibraeva, Alexander Kurovsky, Yana Gummer, Alexey Ruban

**Affiliations:** 1 School of Earth Science & Engineering, Tomsk Polytechnic University, 634050 Tomsk, Russia; makar189@mail.ru (B.M.); pnm1@tpu.ru (P.M.); ruban@tpu.ru (A.R.); 2 Institute of Environmental and Agricultural Biology (X-BIO), University of Tyumen, 625003 Tyumen, Russia; 3 Department of Stratigraphy and Paleontology, University of Granada, 18071 Granada, Spain; 4 Department of Plant Physiology and Biotechnology, Biological Institute, Tomsk State University, 634050 Tomsk, Russia; a.kurovskii@yandex.ru (A.K.);

**Keywords:** glauconite, ammonium dihydrogen phosphate, controlled-release fertiliser, potassium, chemical activation, mechanochemical activation

## Abstract

This paper studies the chemical and mechanochemical preparation of glauconite with ammonium dihydrogen phosphate (ADP) nanocomposites with a ratio of 9:1 in the vol.% and wt.%, respectively. The methods include X-ray diffraction analysis, scanning electron microscope with energy-dispersive X-ray spectroscopy, transmission electron microscopy, infrared spectroscopy, and differential thermal analysis with a quadruple mass spectrometer. The manufactured nanocomposites keep the flaky glauconite structure. Some glauconite unit structures have been thickened due to minimal nitrogen (ammonium) intercalation into the interlayer space. The globular, granular, or pellet mineral particles of nanocomposites can be preserved via chemical techniques. Globular and micro-aggregate particles in nanocomposites comprise a thin film of adsorbed ADP. The two-step mechanochemical method makes it possible to slightly increase the proportion of adsorbed (up to 3.2%) and intercalated (up to 6.0%) nutrients versus chemical ways. Nanocomposites prepared via chemical methods consist of glauconite (90%), adsorbed (1.8–3.6%), and intercalated (3.0–3.7%) substances of ADP. Through the use of a potassium-containing clay mineral as an inhibitor, nitrogen, phosphorus, and potassium (NPK), nanocomposite fertilisers of controlled action were obtained. Targeted and controlled release of nutrients such as phosphate, ammonium, and potassium are expected due to various forms of nutrients on the surface, in the micropores, and in the interlayer space of glauconite. This is confirmed via the stepwise dynamics of the release of ammonium, nitrate, potassium, and phosphate from their created nanocomposites. These features of nanocomposites contribute to the stimulation of plant growth and development when fertilisers are applied to the soil.

## 1. Introduction

Today, sustainable agriculture is crucial to providing the growing global population with sufficient food [1,2,3,4]. To achieve this goal, developing new types of fertilisers and technologies for their use aims to improve the efficiency of modern and future agriculture [5,6,7]. However, using traditional fertilisers such as urea or carbamide, due to their high solubility and low thermal stability, are capable of causing a short-term increase in agricultural production, while their uncontrolled use leads to risks of environmental damage [8,9,10,11]. A representative example is the analysis of the environmental state in areas of developed agriculture, where greenhouse effects [12], eutrophication of water bodies [13], and other negative consequences of the use of traditional nitrogen fertilisers have been identified.

Due to the limited phosphate mineral base, phosphate fertilisers are used less than nitrogen fertilisers [14,15]. On the other hand, sometimes, the production of phosphorus fertilisers from phosphate rocks leads to the pollution of the environment by radioactive by-products and heavy metals [16]. In addition, many mineral phosphate fertilisers exist in unavailable forms [17]. Therefore, using mineral phosphate fertilisers can lead to inefficient use of available phosphates by plants. For improved plant nutrition, synthetic phosphate substances such as ammonium phosphates are applied [18]. The long-term effect of ammonium phosphate is achieved via its encapsulation in the membranes of various substances [19,20,21,22,23,24]. Among the new types of fertilisers are slow-release or controlled-release fertilisers [7,25,26,27,28,29,30].

Controlled-release fertilisers (CRF) are usually complex materials consisting of substances with separated functions [5,25,31]. The key components of CRF are nutrients (often ammonium or phosphates) and inhibitory substances [27,32,33]. Various polymers [25,34,35,36,37], organic materials [22,28,38,39,40], minerals [41,42,43,44,45,46], and heterogeneous substances [44,47,48,49,50,51] can inhibit nutrients in the CRF. Clay minerals (phyllosilicates) are common inorganic materials in the creation slow-release fertilisers [41,45,52,53,54,55,56,57,58,59,60,61,62]. Clay minerals and clay-based complex materials are also used to purify water from phosphate and ammonium ions [63,64]. Phyllosilicates such as vermiculite, montmorillonite, kaolinite, halloysite, and sepiolite are considered relatively inexpensive and environmentally friendly adsorbents of different ions from polluted waters [65]. Glauconite is a potassium-containing dioctahedral phyllosilicate [66,67] that has recently been examined as a urea inhibitor in CRF [43,57]. At the same time, glauconite and glauconite rocks improve soil fertility and plant growth [46,68,69,70,71,72,73,74], making it possible to evaluate them as promising composite materials in the creation of modern multifunctional fertilisers.

The research aimed to prepare glauconite–phosphate nanocomposites using the methods of chemical and mechanochemical activation, with an assessment of the relationship between the mineral as a multifunctional inhibitor and ammonium dihydrogen phosphate as a nutrient.

## 2. Materials and Methods

### 2.1. Materials

This research used a glauconite sample (Karin deposit, Russia) as an inhibitor. The glauconite sample is composed of 70 wt.% glauconite and a 30 wt.% mixture of quartz and feldspars. The average chemical composition of glauconite is as follows [75]: 44.6–56.5 wt.% SiO_2_; 3.5–11.4 wt.% Al_2_O_3_; 17.5–29.8 wt.% Fe_2_O_3(total)_; 6.5–8.8 wt.% K_2_O; 2.6–4.5 wt.% MgO; m0.6–0.8 wt.% CaO; 0.3–0.4 wt.% Na_2_O; and 1.7–14.5 wt.% loss on ignition (LOI). The typical formula of glauconite, measured via energy dispersive analysis, is as follows: K_0.6-0.8_(Al_0-0.8_Mg_0.3-0.5_Fe_0.9-1.5_)_1.4-2.3_(Si_2.9-4.0_Al_0.0-0.5_)O_10_(OH)_2_nH_2_O.

**Figure 1:**
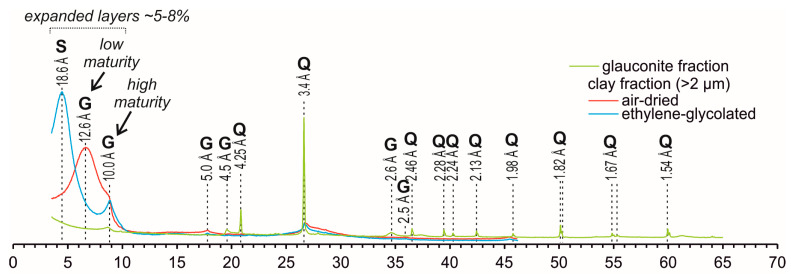
X-ray diffraction patterns of initial glauconite fraction and its oriented clay fractions. G—glauconite; S—smectite; Q—quartz.

XRD patterns of the air-dried clay fraction and ethylene glycol saturation of the initial glauconite sample (Figure 1) display around 5–8% of an expanded smectite phase based on the shift 001 basal peaks from ~12.6 Å to ~18.6 Å [75].

### 2.2. Preparation of Nanocomposites

Ammonium dihydrogen phosphate (ADP; NH_4_H_2_PO_4_) was used as a filling nutrient. ADP consists of more than 15% P_2_O_5_ and 4% nitrogen. The process for creating nanocomposites using glauconite and ADP was executed through four distinct methods: (1) a one-step chemical method; (2) a two-step chemical method with the sodium carbonate; (3) a two-step mechanochemical method of ring milling; and (4) a two-step mechanochemical method of planetary milling. The one-step chemical method involves impregnating the globular glauconite fraction (90 g) in the ADP solution–gel (10 mL) for 48 h until a dry state is reached. The two-step chemical method includes the preliminary impregnating of the globular glauconite fraction (90 g) by the extender (Na_2_CO_3_) and the subsequent activating of the ADP solution–gel (10 mL) for 48 h. The final nanocomposites prepared via the one-step and two-step chemical methods were called Gko90P10 and GkNa90P10, respectively. Mechanochemical activation is distinguished from chemical activation in that it involves grinding glauconite in various mills without employing solutions in the initial stage. The mechanochemical method was subdivided into two variants. Each variant was composed of two steps. The first step is activating the glauconite fraction via ring or planetary milling. The ring milling operation was conducted by a ROCKLABS Standard Ring Mill (with a rotation frequency of 700 rpm and a mass ratio of powders to grinding bodies of 1:5) for 10 min. The planetary milling operation was carried out by AGO-2 with a 1:5 ratio of powder to grinding bodies and a rotation frequency of 1820 rpm for 1 min. The second step is soaking the activated glauconite fraction (90 g) in the ADP solution–gel (10 mL) for 48 h until a dry state is reached. The final nanocomposites produced via two-step mechanochemical methods of ring or planetary milling were named Gka90P10rm and Gka90P10pm, respectively.

### 2.3. Nanocomposite Analysis

The fabricated nanocomposites underwent a series of analyses using X-ray diffraction analysis (XRD), scanning electron microscopy with energy dispersive X-ray spectroscopy (SEM–EDS), transmission electron microscopy (TEM) with selected area electron diffraction (SAED), Fourier transform infrared spectroscopy (FTIR), and differential thermal analysis (thermogravimetric analysis and differential scanning calorimetry, TG–DSC) paired with a quadruple mass spectrometer (MS).

Before and after the nanocomposites’ activation, glauconite structural changes were evaluated. The crystal composition of nanocomposites was identified using a Bruker D2 Phase X-ray diffractometer (Billerica, MA, USA). It used Cu Kα radiation with a voltage of 30 kV and a current setting of 10 mA. Separated fractions with less than 10 μm in size were scanned from 4° to 70° 2-Theta at a step increment of 0.02° with a scan pace of 1.5 s/step. Furthermore, the clay portion with less than 2 µm in size from all nanocomposites, as well as the initial one, was separated then suspended in distilled water, settled for 8–16 h, and then air dried on glass panels. Each sample was assessed in the air-dried and ethylene glycol-solvated states after 24 h in a desiccator at roughly 60 °C. Methods described in [76,77] were used to identify glauconite and smectite. Quantitative mineral evaluation was accomplished using Rietveld analysis [78] with PDXL and Siroquant software packages [79].

The nanocomposites were investigated using the TESCAN VEGA 3 SBU scanning electron microscope (Brno, Czech Republic) and an OXFORD X-Max 50 energy-dispersive adapter (High Wycombe, UK). The SEM–EDS scanning was conducted at a specimen current of 3–12 nA, a spot diameter of ~2 µm, and an accelerating voltage of 20 kV.

The JEOL JEM-2100F transmission electron microscopy (TEM, Tokyo, Japan) at the Center for Sharing Use “Nanomaterials and Nanotechnologies” of Tomsk Polytechnic University was employed to observe crystal structure of glauconite. Specimens were prepared by drying a nanocomposite droplet on a carbon-film-coated copper grid. The TEM analysis was carried out at 200 kV.

The FTIR spectrometer (Shimadzu FTIR 8400S, Kyoto, Japan) chartered the spectra of the nanocomposites between 4000 and 400 cm^−1^ with KBr pellets, a resolution of 4 cm^−1^. This was conducted to ascertain the nanocomposites’ chemical bond functional groups.

Using a STA 449 F5 Jupiter (NETZSCH, Selb, Germany) micro-thermal analyser, the TG–DSC curves were plotted across temperatures ranging from 30 to 1000 °C in an argon setting with a heating rate of 10 °C/min. This facilitated the quantification of the weight ratio of different forms of adsorbed and intercalated substances and a study into thermal degradation. The TG–DSC–MS analysis was realised using a Netzsch TA Quadrupole Mass Spectrometer (QMS) 403C Aeolos for quantification and identification of the evolved gases. Calibration was undertaken just before testing, and the mass spectrometer functioned in electron impact ionisation mode, monitoring ions for the mass-to-charge (*m*/*z*) ratios from 1 to 50.

### 2.4. Leach Testing

Surface soil was collected from agricultural lands in the Tomsk region of Western Siberia (Russia). After root extraction and room temperature drying, the soil was sieved to under 2 mm. A total of 80 g of this dried soil was combined with either nanocomposite in a PVC tube at a dose equivalent to 380 mg N∙kg^−1^. Specific quartz sand quantities were added atop each soil column, and the experiment involved three replications per tested item (three replicates of each sample were called a “plot”).

Soil moisture was retained at 75%. On days 1, 4, 7, 14, 21, 28, 42, and 56, each soil column received 200 mL of deionised water, and the resultant leached solutions (the filtrates) were collected for analysis. The pH, potassium (K^+^), ammonium (NH_4_^+^), and nitrate (NO_3_^−^) concentrations of filtrates were determined using the ionometric method, and phosphate (P_2_O_5_) concentrations were measured using spectrophotometric techniques. Resultant plot values were calculated as the average of three replicates. The cumulative release curves of potassium (K^+^), ammonium (NH_4_^+^), nitrate (NO_3_^−^), and phosphate (P_2_O_5_) were calculated for interpretation.

### 2.5. Plant Cultivation Test

In-lab growth experiments for agricultural plants were performed via the addition of nanocomposites. Soil without fertilisers was used as a control and rationing plot. Weakly acidic agricultural soil (pH 5.1) with 4% organic carbon was used for the tests. Oat seeds (*Avéna satíva*) were cultivated under room conditions for 20 days. Dry nanocomposites were introduced to the soil at 50 kg (potassium) per ha. All tests were conducted thrice (one plot). Germination was estimated after 4 days as the ratio of sprouts to the number of seeds sown. After 20 days, measurements for plant height and weight were undertaken. Upon weight stabilisation, the plants’ dry yield was gauged.

## 3. Results

### 3.1. Mineralogical and Morphological Characterisations of Nanocomposites

**Figure 2:**
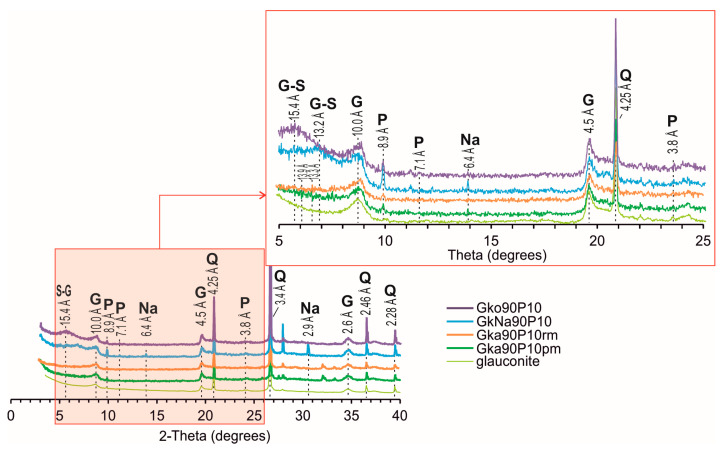
X-ray diffraction patterns of nanocomposites and glauconite fraction. G—glauconite; Na—sodium carbonate; P—ADP substances; Q—quartz.

On XRD patterns, the added ADP is characterised by major reflections at 8.9, 7.1, and 3.8 Å (Figure 2). The intensity of these ADP peaks for GkNa90P10 is slightly higher than for other nanocomposites. The mineral composition in the nanocomposites is represented by the glauconite with quartz. The XRD pattern of glauconite shows reflections at 10.0, 4.5, and 2.6 Å. Reflections at 4.2, 3.4, 2.5, and 2.3 Å identify quartz. In addition, the XRD pattern of GkNa90P10 displayed reflections of the sodium carbonate at 6.4 and 2.9 Å (Figure 2). In glauconite, there is shift of the first basal reflection (001) in the direction of an increase in the interplanar distance up to 13.2–15.4 Å for nanocomposites produced via the chemical method, and up to 13.3–13.9 Å for nanocomposites produced via the mechanochemical process.

**Figure 3:**
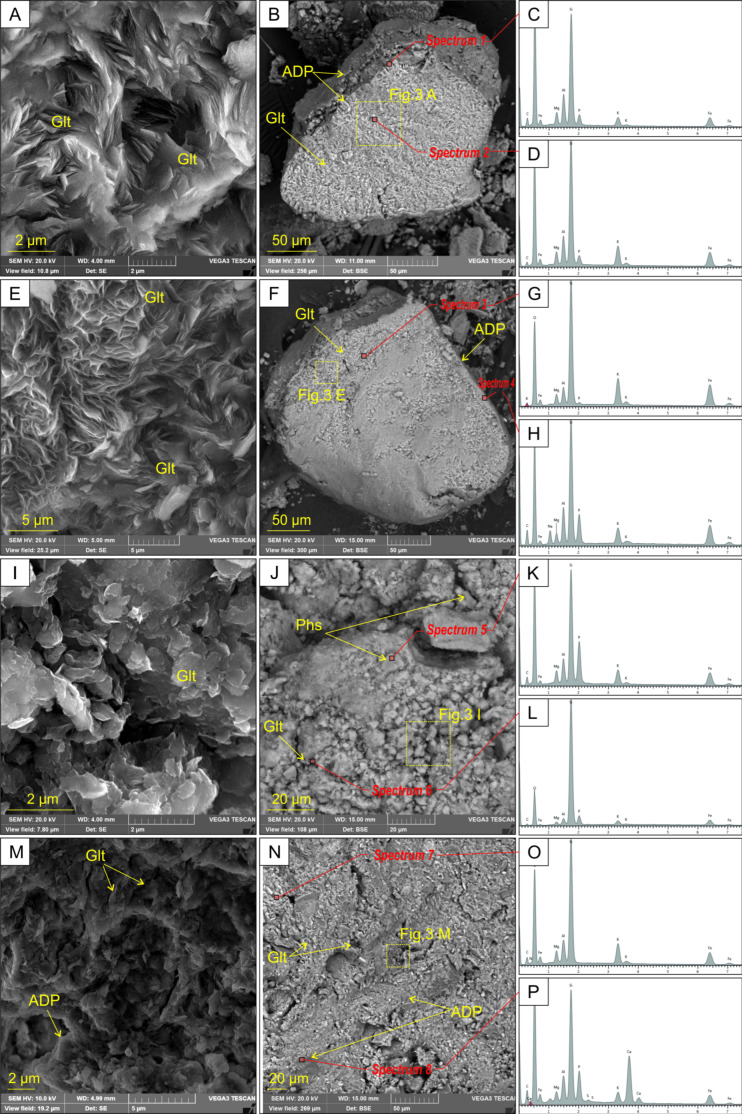
High-resolution SEM images, overview SEM images, and representative EDS spectra of nanocomposites: (**A**–**D**) Gko90P10; (**E**–**H**) GkNa90P10; (**I**–**L**) Gka90P10rm; (**M**–**P**) Gka90P10pm. SEM images show glauconite original flakes (**A**,**E**), glauconite deformed flakes (**I**,**M**), pellets (**B**,**F**) and microparticles (**J**,**N**) covered with a thin film with a high proportion of phosphorus (**C**,**H**,**K**,**P**) according to EDS analysis. The covering film of glauconite pellets contains up to 6.9% phosphorus (**D**,**H**). Glt—glauconite; ADP—ADP substances.

The morphology of the chemically prepared Gko90P10 and GkNa90P10 nanocomposites retain their relic, globular, pellet glauconite grains with 100–400 µm in diameter and a characteristic internal structure (Figure 3A,B,E,F). Glauconite flakes are covered with a thin film of ADP of 0.4–2 µm thick. According to the EDS analysis, glauconite of Gko90P10 contains 5.3–8.1% potassium and 0.9–2.2% phosphorus of the covering thin film. The surface of the pellets is covered with ADP film with up to 6.9% of phosphorus (Figure 3H) and thickness up to 2–8 µm.

Microparticles represent the morphology of the mechanochemically produced Gka90P10rm and Gka90P10pm nanocomposites with sizes of 40–120 µm in diameter and a weakly preserved glauconite structure (Figure 3I,J,M,N). The structure of glauconite microparticles is represented by modified flakes less than 4 µm in size. The surface of the microparticles is covered with ADP films and aggregates (Figure 3). The microparticles are composed of phosphorus of 2.0–10.1%, potassium of 3.6–7.8%, and nitrogen of up to 2.3%.

### 3.2. Crystal Structure Characteristics of Nanocomposites

**Figure 4:**
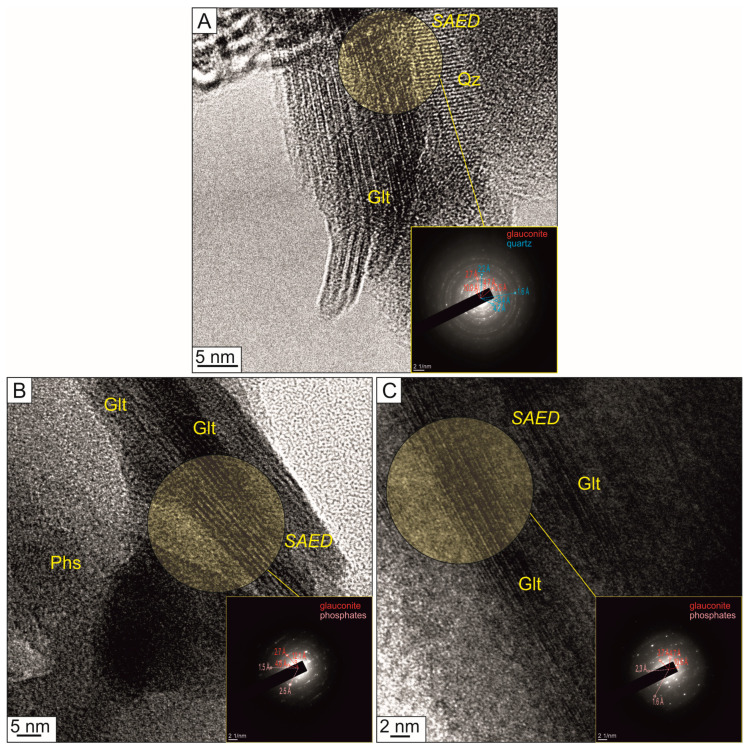
High-resolution TEM images of clay particles and SAED patterns with the main interpreted interplanar spaces of the original glauconite with impurities of quartz crystals (**A**), the produced Gko90P10 (**B**), and Gka90P10rm (**C**) nanocomposites. Glt—glauconite; Phs—phosphate crystals; Qz—quartz.

Glauconite particles comprise nano-scale spindles of mica crystallites with a layered structure on high-resolution TEM images of the original mineral and activated nanocomposites (Figure 4). The glauconite unit structure consists of tetrahedral, octahedral, and tetrahedral sheets and an interlayer. The unit structure thickness of the original glauconite (Figure 4A) is 9.9–11.5 Å (average 10.8 Å), in which the interlayer thickness varies within a range of 1.9–3.0 Å (average 2.5 Å). The mineral particles of nanocomposites prepared via the chemical methods (Figure 4B) contain glauconite spindles and phosphate nanocrystals. The glauconite unit structure of Gko90P10 and GkNa90P10 obtained via TEM images ranges from 9.3 to 14.7 Å (average 11.9 Å), in which the interlayer thickness is 2.4–4.2 Å (average 3.2 Å). The unit structure of the Gka90P10rm (Figure 4C) and Gka90P10pm nanocomposites varies from 9.7 to 13.4 Å (average 11.2 Å) with a 1.9–3.6 Å (average 3.0 Å) micro-thickness of the interlayer.

### 3.3. Chemical Characterisations of Nanocomposites

**Figure 5:**
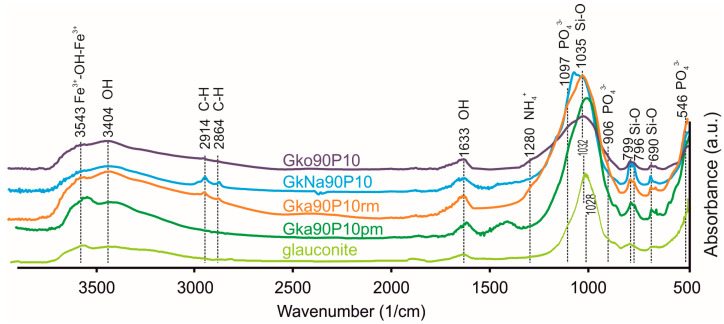
FTIR-spectra of nanocomposites and glauconite. The spectra of the nanocomposites differ in the presence of peaks for PO_4_^3−^, NH_4_^+^, CH, and OH ions. All spectrums reflect Si–O and Fe^3+^–OH vibrations of glauconite.

All FTIR spectra show vibrational bands related to the PO_4_^3-^ phosphate ion (Figure 5); that is, bending vibrations at 546 cm^−1^ (O–P–O), gentle asymmetric vibrations at 906 cm^−1^, 1097 cm^−1^ (P–O–H). The peaks at 796 and 799 cm^−1^ show Si-O stretching vibrations in the tetrahedral sheet of the glauconite. A 1035 cm^−1^ peak characterises symmetric vibrations of Si–O of glauconite. Asymmetric deformation vibrations of N–H in NH_4_^+^ ions are reflected at 1280 cm^−1^. Stretching vibrations of CH_2_ are observed in the 2864–2914 cm^−1^. Asymmetric bending vibrations of OH^-^ ions in the octahedral positions of the mineral are reflected in the 3543 cm^−1^ bands. The CH_2_ peaks at the range of 2864–2914 cm^−1^, as well as the PO_4_^3−^ peak at the 1097 cm^−1^ and the NH_4_^+^ peak at the 1280 cm^−1^, are more intense in GkNa90P10 and Gka90P10rm than in Gko90P10 and Gka90P10pm.

**Figure 6:**
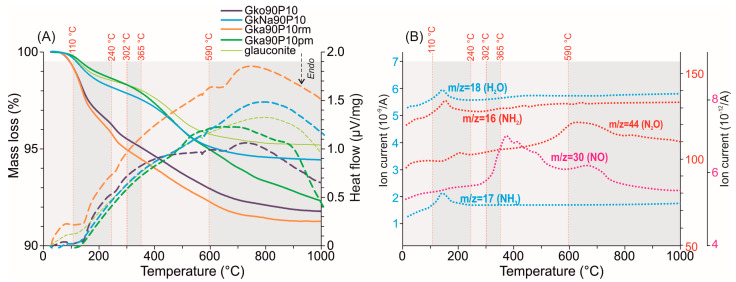
(**A**) TGA shown as continuous lines and DSC depicted with dotted lines for both nanocomposites and glauconite. (**B**) Chosen representative MS multi-ion detection curves (*m*/*z* = 17 (NH_3_), 18 (H_2_O), 30 (NO), and 44 (N_2_O)) for Gko90P10 nanocomposite.

According to the TG analysis (Figure 6A, Table 1), six main stages of weight loss in the prepared nanocomposites are distinguished. Removal of free water by 0.9 wt.% occurs in the temperature range of 0–110 °C. In the range of 110–240 °C, weight losses are recorded at 1.1–3.2 wt.%, more than the original glauconite fraction. Perhaps these weight losses are associated with the release of adsorbed phosphates and water. Some ions, such as NH_3_, NH_2_, and H_2_O, are observed in this temperature interval according to QMS analysis (Figure 6B). The maximum ion current intensity is typical for H_2_O and NH_3_. A weight loss of 0.4–0.9 wt.% at the 240–302 °C stage indicates the removal of adsorbed substances with a QMS peak of N_2_O (Figure 6B). The weight losses in 302–365 °C and 365–590 °C vary in nanocomposites in the range of 2.5–3.3 wt.%, which are associated with the removal of interlayer solvating water from the mineral particles. The release of NO and N_2_O is detected in this range (Figure 6B). An acute endothermic effect at 572 °C corresponds to the quartz impurity. Weight loss in the range of 590–1000 °C by 0.7–2.7 wt.% in nanocomposites indicates dehydroxylation of glauconite. The DSC curves of the nanocomposites show a gentle endothermic effect at 648 °C in this interval. In the original glauconite, a weight loss of 0.6 wt.% is recorded in 590–1000 °C without endothermic effect. In this temperature interval, a weak ion current intensity of N_2_O and NO is noted (Figure 6B).

### 3.4. Kinetic Nutrients Releases

**Figure 7:**
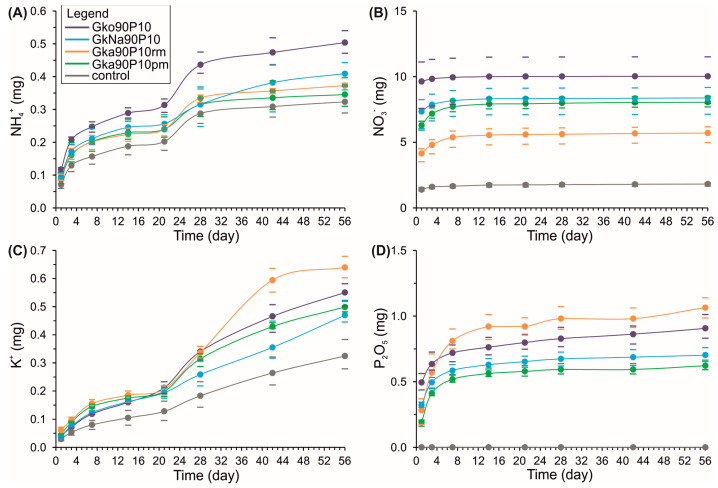
Cumulative curves of ammonium (**A**), nitrate (**B**), potassium (**C**), and phosphate (**D**) releases from nanocomposites glauconite–ADP, contrasted with a reference plot devoid of nanocomposites from lab experiments. Dashes show minimum and maximum cumulative values. The statistical significance of every parameter corresponds to the *p* = 0.05 level. Bars represent minimal and maximal values.

The laboratory tests revealed varied leaching kinetics for ammonium, nitrate, potassium, and phosphate in the created glauconite–ADP nanocomposites. Observing ammonium (Figure 7A), two peak releases periods were identified: days 1st–7th and 21st–28th. Between days 7 and 21, ammonium is released slowly. Moreover, after day 28, ammonium has prolonged mobilisation kinetics, except for the GkNa90P10 nanocomposite. Ammonium release from the GkNa90P10 nanocomposite increases linearly from day 21 to day 56. The best kinetics of ammonium release was observed for the nanocomposites prepared chemically. Nitrates showcased a notable release rate within the initial seven days across all nanocomposites (Figure 7B). This was followed by a minimal leaching rate of 0.2–0.3 mg from days 7 to 56. Maximum nitrate release was observed in the chemically activated nanocomposites. Considering potassium release (Figure 7C), it experienced three to four primary stages that deviated from the reference plot. In all nanocomposites, the first two stages are synchronously recorded. The initial stage, culminating on the 7th day, marked a rapid release rate. The subsequent phase recorded a subdued release from the 7th to the 21st day. After day 21 of the soil column leaching experiments, the kinetics in different nanocomposites have some differences: in the nanocomposites prepared via chemical methods, the potassium dynamics are described by a consistent linear increase from day 21 to day 56; mechanochemically activated nanocomposites (Gk90P10rm and Gk90P10pm) release potassium in two steps during this period. The period from day 21 to day 43 showcased peak kinetics, while after the 43rd day, the release rate stabilised. The phosphate dynamics are maximal in the first 14 days of leaching, after which it has a prolonged character with low release levels (Figure 7D).

### 3.5. Influence of Nanocomposite on Plant Growth

**Figure 8:**
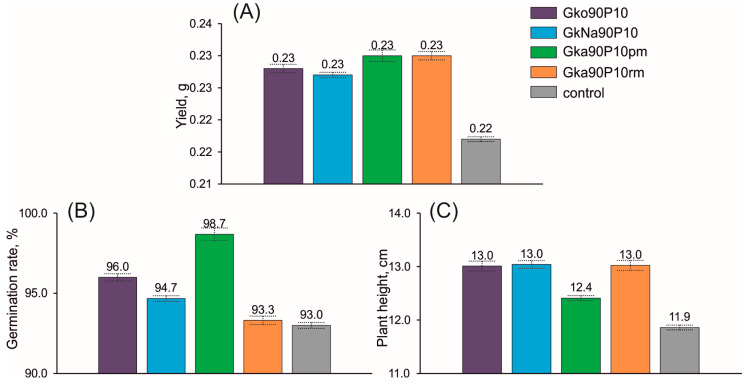
Dry yield (**A**), germination rate (**B**), and plant height (**C**) of oat (*Avéna satíva*) due to application studied glauconite-ADP nanocomposite fertilisers based on the laboratory experiment. The statistical significance of every parameter indicates at the *p* = 0.05 level. Bars represent standard deviations.

The dry oat yield (*Avéna satíva*) increased in all variants of the experiments with the tested nanocomposites relative to the control (Figure 8). The dry weight (yield) changed in a range of 0.227–0.230 g for all plots, corresponding to 0.217 g for the control sample (Figure 8A). The yield in the plot with applied nanocomposites from glauconite–ADP mixtures increased by more than 4.6%. The germination rate (Figure 8B) when using nanocomposites varies in the range of 93.3–98.7%, while in the control plot (without fertilisers), this indicator is 93.0%. The maximum germination energy was noted for nanocomposites prepared mechanochemically in a planetary mill. The average plant height (Figure 8C) in the experiments with nanocomposites varied from 12.4 to 13.0 cm compared to 11.9 cm for the control sample. This corresponds to an increase in plant height of 4.6–9.9% when using nanocomposites.

## 4. Discussion

### 4.1. Characteristics of Glauconite–ADP Nanocomposites

The short shift in the slight basal reflection (001) in the bulk samples of nanocomposites (Figure 2) indicates that a significant part of the ADP was adsorbed on the basal interlayer planes of the mineral particles. This is confirmed by increased phosphorus content in the mineral aggregates (Figure 3C,D,G,H). In the Gka90P10rm and Gka90P10pm nanocomposites, the covering thin film on glauconite flakes contains up to 10.1% of the phosphorus (Figure 3K), which displays a slightly higher intensity of ADP peaks in the XRD pattern (Figure 2) and the FTIR spectrum (Figure 5) as the result of an increased adsorbed ADP relative to the Gko90P10 nanocomposite (Figure 3D). The preservation of the globular shape of the glauconite in the Gko90P10 and GkNa90P10 nanocomposites (Figure 2) is an advantage for their use as mineral fertiliser. The granular form improves the mechanical–physical properties of the soil and also allows for efficient fertilisation [7].

The unit structure thickness of some glauconite particles before and after chemical activation, according to the TEM data with SAED, varies from 9.9–11.5 Å to 9.3–14.7 Å (Figure 4), which indicates a slight expansion of the mineral crystal lattice, probably due to weak intercalation of ammonium into the interlayer of the mineral unit structure [41]. This is also reflected in the increase in the interlayer thickness from 1.9–3.0 Å to 2.4–4.2 Å. An insignificant part of NH_4_^+^ from ADP was most likely intercalated into the expanding structures of smectite layers in glauconite [75].

Deformation vibrations of phosphate ions are manifested in the FTIR spectra of nanocomposites (Figure 5) prepared via both chemical and mechanochemical techniques. Gentle asymmetric P–O–H oscillations at 906 cm^−1^ and 1097 cm^−1^ [80,81] are associated with the adsorbed part of the ADP on the mineral particles, including the edges of crystal basal surfaces. The intensity of these oscillations depends on the amount of ADP, which is connected with glauconite particles. The symmetric deformation vibrations of NH_4_^+^ indicate the intercalation of ammonium into the vacant space of the glauconite or smectite interlayer. Adsorbed ADP substances and intercalated ammonium will be released with a prolonged effect [25,26,27].

The total weight loss of the nanocomposites (Figure 6, Table 1) is 8.2 wt.%, 5.6 wt.%, 8.7 wt.%, and 7.7 wt.% for Gko90P10, GkNa90P10, Gka90P10rm, and Gka90P10pm, respectively, which is almost comparable to the initial added amount of ADP. The maximum adsorbed ADP varies from 2.8 to 3.2 wt.% for the Gko90P10 and Gka90P10rm nanocomposites, respectively, which is associated with the decomposition of a thin coating on the surface of mineral particles (Figure 3B,F). Adsorbed ADP into the micropore space is estimated at 0.4–0.9 wt.%, including the removal NH_3_, NH_2_, and H_2_O ions. Substances (NO and N_2_O) removal from the mineral interlayer space at 302–590 °C, a sharp endothermic effect at 572 °C, are synchronous for both the nanocomposites and the original glauconite. A gentle endothermic effect at 648 °C and the increased weight loss in nanocomposites relative to the original glauconite (Figure 6, Table 1) at 590–1000 °C prove the release of ADP from the mineral structure [82]. This is probably due to the incorporation of positively charged NH_4_^+^ ions on the edges of the octahedral basal planes of glauconite.

### 4.2. Advantages of Glauconite–ADP Nanocomposites

The prepared nanocomposites have similar features to previously studied complex composites consisting of monoammonium phosphate (or ADP) and kaolinite [83] or smectite [47,84]. However, glauconite, as an inhibitor of phosphates, is used for the first time, and it distinguishes the studied composites from composites with kaolinite or smectite as an inhibitor [41,59,83,85,86].

Nutrient discharges (such as ammonium, nitrate, potassium, and phosphates) present a sequential kinetic, linked to their association with the mineral, as depicted in Figure 7. The sequential patterns of nutrient release reflect their varied concentrations within the nanocomposites. It is postulated that easily accessible forms are tied to adsorbed substances in macro-pores dislodging from the nanocomposites primarily between days 1 to 21 or days 1 to 7, relevant to ammonium, nitrate, and phosphates, respectively. This is followed by a transition whereby adsorbed ammonium is liberated from the meso-pore space between the 21st and 28th days. The interlayered ammonium from the mineral interlayers is extracted by the 28th day of nanocomposite interaction with the soil. Generally, nanocomposites prepared via the mechanochemical method using glauconite–ADP combinations exhibit an added step. The sequential kinetics concerning nutrient leaches, particularly ammonium, potassium, and phosphates, endorse the dual advantageous role of stratified glauconite in nanocomposites, serving both as a deterrent for nutrients and as a potassium reservoir.

As a result of a series of laboratory experiments, it was found that the maximum stimulating effect on plant growth and development was exerted by nanocomposites prepared mechanochemically from mixtures of mineral and ADP in a 9:1 ratio (Figure 8). All plots with the investigated nanocomposite fertilisers demonstrated a stable positive effect on the growth and development of oats.

**Figure 9:**
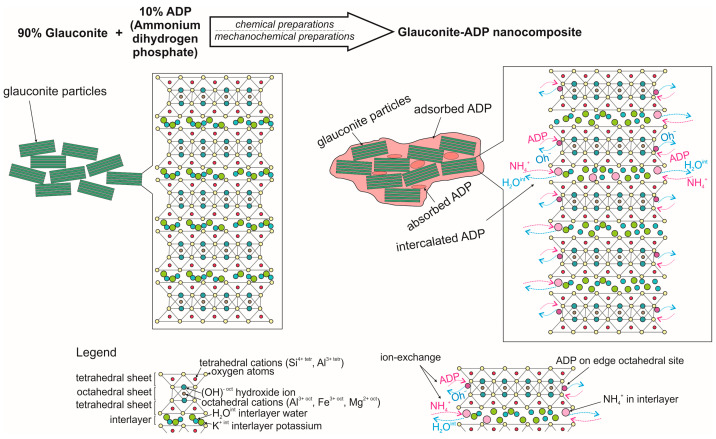
Schematic model of interactions between glauconite (90%) and ADP (10%) in the mechanochemical or chemical preparations of nanocomposites.

Glauconite–ADP nanocomposites prepared via chemical and mechanochemical methods have general advantages (Table 2). The interaction between glauconite and ADP at a ratio of 9:1 made it possible to create nanocomposites with four main types of ammonium phosphate nutrient: adsorbed in macro- and mesopore space; intercalated in the smectite interlayer; or adsorbed on the edge basal planes of mineral particles (Figure 9). Some essential portion of the NH_4_^+^ ions is intercalated into the interlayer space. The created nanocomposites, in addition to phosphates and ammonium, contain exchangeable potassium, which allows them to be evaluated as NPK fertilisers. Different forms of ADP in the nanocomposites and interlayer potassium will provide a controlled release of nutrients to plants. The chemical method allows for the preservation of the functional globular form of glauconite, while the two-stage mechanochemical method leads to a slight increase in the adsorption of ADP on the active surfaces of the mineral and intercalation of ammonium in interlayer of glauconite. The use of glauconite–ADP mixtures, for example, compared to glauconite–carbamide mixtures [75], has the benefit of creating complex NPK nanocomposite controlled-release fertilisers. Nanocomposite fertilisers derived from glauconite–ADP mixtures are particularly recommended for organic farming. This mineral, when used independently as an eco-friendly addition, enhances soil quality [72].

## 5. Conclusions

The study of the chemical and mechanochemical activation of glauconite–ADP nanocomposites at a ratio of 9:1 made it possible to draw the following conclusions.

The nanocomposites reflect a glauconite structure without significant expansion of the interplanar spaces of the mineral upon activation with ADP. The unit structure thickness of some elementary mineral particles increases due to the minimal intercalation of ammonium into the interlayer of glauconite.

The chemical method of preparing nanocomposites allows for obtaining substances in a preserved glauconite form. Globular and micro-aggregate particles in nanocomposites are covered with a thin film of adsorbed ADP. The mechanochemical method slightly increases the proportion of adsorbed (up to 3.2 wt.%) and intercalated (up to 6.0 wt.%) ADP.

By using a potassium-containing clay mineral as an inhibitor, complex NPK nanocomposite fertilisers with controlled action were obtained. Targeted release of nutrients is expected due to various forms of nutrients on the surface, in the micropores, in the interlayer space, and in the edge basal planes of glauconite. This is confirmed via the stepwise dynamics of the release of ammonium, nitrate, potassium, and phosphate from created nanocomposites.

Applying nanocomposites to soils as fertiliser stimulates an increase in yield, germination rate, and plant height.

## Figures and Tables

**Table 1:** Mass reduction data for glauconite–ADP nanocomposites in comparison with initial glauconite as per TGA.

	Nanocomposites	Weight Loss Intervals (°C)					
		0–110	110–240	240–302	302–365	365–590	590–1000
Weight loss (%)	Gko90P10	99.1	96.3	95.5	95.0	93.0	91.8
	GkNa90P10	99.6	98.2	97.9	97.4	95.2	94.4
	Gka90P10rm	99.1	95.9	94.9	94.4	92.3	91.3
	Gka90P10pm	99.8	98.7	98.3	97.8	95.0	92.3
	Glauconite	99.7	98.6	98.3	97.9	95.8	95.2
Final residue (%)	Gko90P10	0.9	2.8	0.8	0.5	1.9	1.2
	GkNa90P10	0.4	1.4	0.4	0.4	2.3	0.7
	Gka90P10rm	0.9	3.2	0.9	0.6	2.0	1.1
	Gka90P10pm	0.2	1.1	0.4	0.5	2.7	2.7
	Glauconite	0.3	1.1	0.3	0.4	2.1	0.6

**Table 2:** Comparative features of glauconite–ADP nanocomposites.

Features	Nanocomposites			
	Gko90P10	GkNa90P10	Gka90P10rm	Gka90P10pm
A shift of the basal reflection 001 on the bulk XRD	Up to 15.4 Å	Up to 13.2 Å	Up to 13.3 Å	Up to 13.9 Å
Morphology of elementary particles	The relic globular and pellet grains with size of 100–400 µm		Microparticles with size of 40–120 µm	
ADP coating	2–8 µm thick film		Film and aggregates	
Mineral unit structure	The unit structure thickness is 9.3–14.7 Å (average: 11.9 Å)		The unit structure thickness is 9.7–13.4 Å (average: 11.2 Å)	
	The interlayer varies from 2.4 to 4.2 Å (average: 3.2 Å)		The interlayer varies from 1.9 to 3.6 Å (average: 3.0 Å)	
Adsorbed ADF in interparticle space (macropores)	2.8 wt.%	1.4 wt.%	3.2 wt.%	1.1 wt.%
Adsorbed ADF on edge plane (mesopores)	0.8 wt.%	0.4 wt.%	0.9 wt.%	0.4 wt.%
Intercalated NH_4_^+^	3.7 wt.%	3.4 wt.%	3.7 wt.%	6.0 wt.%

## Data Availability

Data is contained within the article.
